# 2019 Ambon aftershocks catalogue data compiled using local and regional seismic networks

**DOI:** 10.1016/j.dib.2021.106728

**Published:** 2021-01-09

**Authors:** David P. Sahara, Andri D. Nugraha, Abdul Muhari, Andi Azhar Rusdin, Shindy Rosalia, Awali Priyono, Z. Zulfakriza, Sri Widiyantoro, Nanang T. Puspito, Aditya Lesmana, Dian Kusumawati, A. Ardianto, Aria Widhi Baskara, Yehezkiel Halauwet, Hasbi Ash Shiddiqi

**Affiliations:** a Global Geophysics Research Group, Institut Teknologi Bandung, Bandung, Indonesia; b National Disaster Management Authority of Indonesia, Jakarta, Indonesia; c Agency for Meteorology, Climatology and Geophysics of Indonesia, Jakarta, Indonesia; d Faculty of Engineering, Maranatha Christian University, Bandung, Indonesia; e Geophysical Engineering Study Program, Faculty of Mining and Petroleum Engineering, Institut Teknologi Bandung, Bandung, Indonesia; f Department of Earth Science, University of Bergen, Allègaten 41, N-5007 Bergen, Norway

**Keywords:** 2019 Ambon earthquake, Aftershocks, Seismic catalogs, Local seismic network

## Abstract

This article presents earthquake catalogs of the 2019 Ambon aftershocks compiled from regional the Agency for Meteorology, Climatology, and Geophysics of Indonesia (BMKG) and local seismic networks deployed in [1]. The final earthquake catalogs are comprised of 1009 and 1764 aftershocks compiled from regional [2] and local network [1], respectively, which span the period of October 18th to December 15th, 2019. The range of their spatial region is −3.70^o^ to −3.30^o^ on the latitude and 128.15^–^128.60^o^ on the longitude. Additionally, focal mechanism solutions of the main Mw 6.5 and its biggest triggered aftershock Mw 5.2 events were acquired. Such datasets could provide a basis for further seismology analysis, including seismic tomography, source mechanism analysis, and further seismic hazard analysis in the Ambon and Seram islands. This paper and its dataset are a companion for a published article in the Tectonophysics under the title “Source Mechanism and Triggered Large Aftershocks of the Mw 6.5 Ambon, Indonesia Earthquake” [1].

## Specifications Table

**Table:** 

Subject	Earth and Planetary Sciences (Geophysics)
Specific subject area	Seismic Monitoring
Type of data	Table, Figures
How data were acquired	Coordinates of seismographic station locations were compiled from and Sahara et al. [1] and the Agency for Meteorology, Climatology, and Geophysics of Indonesia (BMKG) database [2]. Velocity model was taken from AK135 [3] and Sahara et al. [1]. Aftershock hypocenter catalogs were taken from Sahara et al. [1] and BMKG [2]. Focal mechanism data were obtained from the following published sources: The Global Centroid Moment Tensor (GCMT) [4], United States Geological Survey (USGS) [5], and BMKG [2]. The slip model of the main 2019 Ambon earthquake was taken from Sahara et al [1].
Data format	Processed, Analysed
Parameters for data collection	Several parameters were considered during the data collection, i.e. geographic coordinates (longitudes and latitudes), start and end dates.
Description of data collection	Data were collected from several sources including international, regional, and publications.
Data source location	Country: Indonesia Region: Ambon, Seram Island and surroundings Latitude and longitude. The primary data of the hypocenter and focal mechanism catalogs were taken from [1], [2], [3], [4], [5]
Data accessibility	The data files are available on a public repository Repository name: Mendeley Data, v1. Data identification number: http://dx.doi.org/10.17632/z5nmbmmddt.1 Direct URL to data: http://dx.doi.org/10.17632/z5nmbmmddt.1
Related research article	Authors names: D. P. Sahara, A. D. Nugraha, A.Muhari, A. A. Rusdin, Shindy Rosalia, Awali Priyono, Z. Zulfakriza, Sri Widiyantoro, Nanang T. Puspito, Andreas Rietbrock, Aditya Lesmana, Dian Kusumawati, A. Ardianto, Aria Widhi Baskara, Yehezkiel Halauwet, Hasbi Ash Shiddiqi, Muhammad Taufiq Rafie, Raisha Pradisti, Prima Widianto Mozef, M. Zain Tuakia, Erfin Elly Title: Source Mechanism and Triggered Large Aftershocks of the Mw 6.5 Ambon, Indonesia Earthquake Journal: Tectonophysics Status: Published [https://doi.org/10.1016/j.tecto.2020.228709]

## Value of the Data

• It is a catalog of 1075 aftershocks obtained using a local seismic network deployed to infer the fault structures responsible for the 2019 Ambon Mw 6.5 earthquake and its largest Mw 5.2 triggered event.
• Geoscientists who would like to further image the complex fault structures and their seismic coupling in the Ambon and Seram island could benefit from the catalog.
• Aftershock catalogs and main event focal-mechanism solutions serve as very useful indicators for the seismotectonic and the in-situ stress in the region.
• Both earthquake and slip model can be incorporated during the analysis of the fault reactivation potential in the surrounding area.
• Definition of a representative and reliable seismic source model based on such detailed aftershock catalogs and slip model is the most crucial element necessary to achieve appropriate estimates of seismic hazard and risk in the region.

## 1 Data Description

The first dataset contains station coordinates and velocity model for determining the hypocenter location of 2019 Ambon aftershocks. This is a Microsoft Excel worksheet consisting of two sheets. The first sheet contains the coordinates of the eleven local seismographic stations used for monitoring the 2019 Ambon aftershocks [1] and the four regional the Agency for Meteorology, Climatology, and Geophysics of Indonesia (BMKG) [2]. The regional ones are permanent stations while the locals were deployed between October 18th to December 15th, 2019. This sheet organized into five columns, i.e. the three first rows describes the coordinates (Longitude (in^o^), Latitude (in^o^), and Elevation (in m), the fourth is the network (either regional (BMKG) or local) and the last is the record duration of each station (zero for permanent stations). The second sheet includes the velocity model used in locating the aftershocks in [1]. Each row in this sheet depicts the depth of the top layer (in km), the velocity of P and S wave of the global AK135 [3] (in m/s), and the updated velocity model suited local Ambon geological condition (in m/s).

The second Microsoft Excel file in the Mendeley Data repository includes earthquake catalogs of 2019 Ambon aftershocks. The files contain a total of 1009 and 1764 earthquakes taken from BMKG catalog [2] and local network [1], respectively, organized into 13 columns; each row describes a single earthquake while each column describes the related parameters. Since [1] conducted a step-wise approach in determining the hypocenter locations, there are three catalogs resulted from a local study [1], i.e. (i) initial location using nonlinear approach [6,7] and AK135 velocity model [3], (ii) updated joint-inversion location and velocity model using Velest software [8], and (iii) relative relocations using double-difference (DD) concept [9], [10]. The definition of the different parameters (columns) mentioned in this file are the following:

A: YEAR; B: MONTH; C: DAY: date type variables indicating the date for each earthquake.
D: HOUR; E: MINUTE; F: SECOND: date type variables indicating the time for each earthquake.
G: LONGITUDE; H: LATITUDE: double type variables (three decimal digits) indicating the location (longitude and latitude) for each event.
I: DEPTH: double type variable (one decimal digit) indicating the depth of each individual earthquake.
J: MAGNITUDE: double type variables indicating the reported magnitudes (one decimal digit) for the included earthquakes. The type of the magnitude is ML (Local magnitudes) for regional BMKG catalog [2] and Mw (Moment Magnitude) for local catalog [1].
K,L,M: UNCERTAINTY: double type variables indicating the calculated uncertainty (in km for X, Y, and Depth respectively) for each event. This information is only available for aftershock catalog determined using local seismic network [1].

The third file in the repository is the focal mechanism of the 2019 Ambon aftershock main event and its biggest M 5.2 triggered event. The focal solutions of the main M 6.5 event were taken from Global CMT [4], USGS [5], and BMKG [2]. The focal solution of the biggest M 5.2 triggered event was taken from Global CMT [4]. The definition of the columns in the focal mechanism solution is the following:

A–I: similar with the format of the aftershock catalog mentioned earlier.
J: MAGNITUDE: double type variable (one decimal digit) indicating the final considered moment magnitude for each earthquake included.
K: Strike 1; L: Dip 1; M: Rake 1; N: Strike 2; O: Dip 2; P: Rake 2: these columns represent the two nodal planes for the focal mechanism solution for each earthquake; each column contains a number for each mentioned individual parameter (strike, dip, and rake angles).
Q: SOURCE: string variables indicating the source of the focal solutions.

The fourth Microsoft Excel file is the slip model of the main 2019 Ambon earthquake derived using teleseismic data. The teleseismic dataset of the M 6.5 event was gathered from the Incorporated Research Institutions for Seismology (IRIS) Data Management Center (DMC) (http://ds.iris.edu/ds/nodes/dmc) under the network codes II, IU, G, IC, dan RM. This file consists of two tables, i.e. represent each focal solution plane. Each table consists of four columns: X and Y location, seismic moments, and rake of each node. The header describes the reference point (X=0 km and Y=0 km) used in the inversion scheme.

## 2 Experimental Design, Materials and Methods

**Fig 1:**
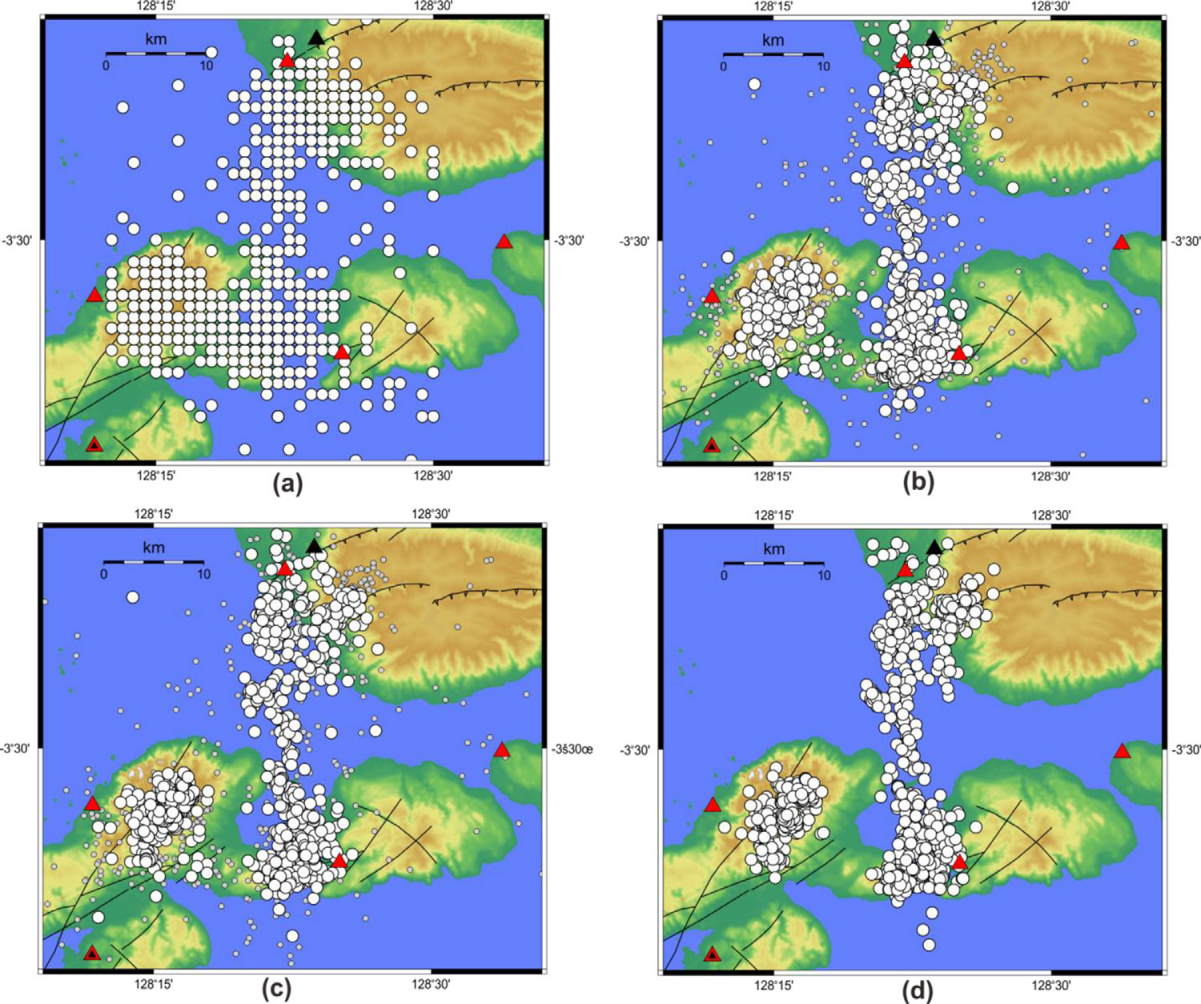
Earthquake catalogs: (a) BMKG catalogs [2]; (b) initial location using nonlinear approach [6,7] and AK135 velocity model [3] plotted for those events with location uncertainties less than 5 km (big white circles) and of more than 5 km (small grey circles); (c)updated joint-inversion location and velocity model using Velest software [8] plotted for uncertainties under 5 km (Big white circles) and uncertainties more than 5 km (small grey circles); (d) relative relocations using double-difference concept [9,10].

2019 Ambon aftershocks located in the spatial region spanning longitudes between 128.15^o^ and 128.60^o^ and latitudes between −3.70^o^ and −3.30^o^ and occurred between October 18th to December 15th, 2019 were considered during the dataset compilation. During this monitoring period, 1009 and 1764 events were compiled from the BMKG [2] and local seismic network catalog [1] (Fig. 1). For the local seismic network data, the locations of aftershock were determined in a stepwise approach, i.e. (i) initial location determination using a non-linear approach [6], [7], (ii) updating the velocity model [8], and (iii) relative DD relocation [9], [10]. The velocity models used for determining the location of aftershock events in each step are presented. Events location and their uncertainty in each step are documented. Moment magnitude was estimated using spectral fitting for each aftershock after DD relocation.

**Fig. 2:**
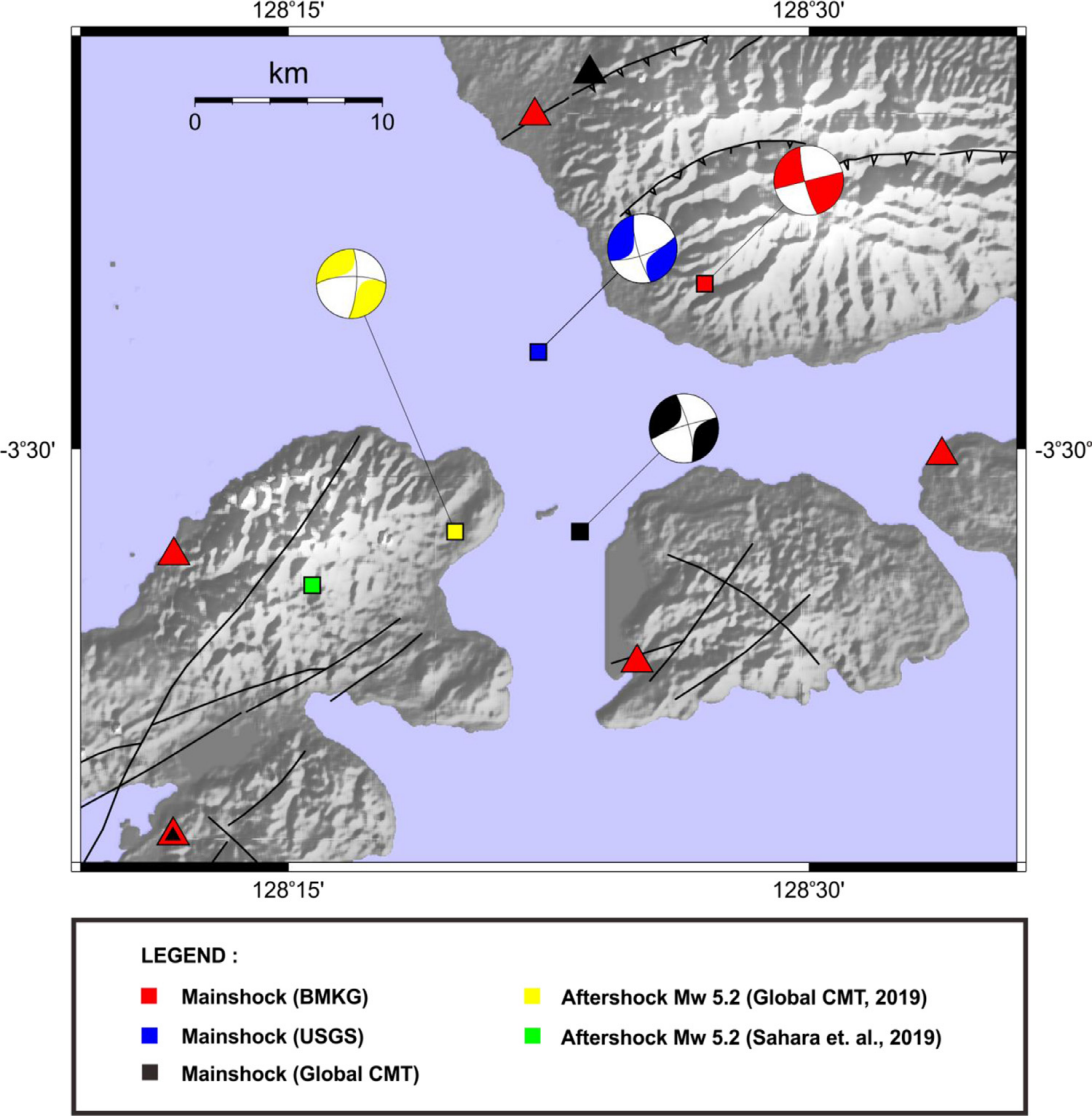
Focal mechanism Solution of the 2019 Ambon aftershock main event and its biggest M 5.2 triggered event. The focal solutions of the main M 6.5 event were taken from Global CMT [4] (black beachball) corresponds to its hypocenter (black rectangle), USGS [5] (blue beachball) corresponds to its hypocenter (blue rectangle) and BMKG [2] (red beachball) corresponds to its hypocenter (red rectangle). The focal solution of the biggest M 5.2 triggered event was taken from Global CMT [4] (yellow beachball) corresponds to its hypocenter (yellow rectangle). The Green rectangle shows the relative DD locations of M 5.2 triggered event taken from [1].

Focal mechanism solutions of the 2019 Ambon main event and its largest M 5.2 triggered event were obtained from gCMT [4], USGS [5], and BMKG bulletins [2] (Fig. 2). Slip inversion was conducted using Teleseismic Body-Wave Inversion Program [11], [12]. Teleseismic waveform data used in the slip inversion were obtained from the Incorporated Research Institutions for Seismology (IRIS) Data Management Center (DMC) [13] under the network codes II, IU, G, IC, dan RM.

The related article has been submitted to the Tectonophysics under the title “Source Mechanism and Triggered Large Aftershocks of the Mw 6.5 Ambon, Indonesia Earthquake”.

## CRediT Author Statement

**DPS, ADN, AM, AAR and SR:** Conceptualization, Methodology, Software; **ZZ, SW, NTP and AP:** Writing-Original draft preparation; **AL, DK, AA, YH and AWB:** Visualization, Software and Validation; **HAS:** Software, Slip Model.

## Declaration of Competing Interest

The authors declare that they have no known competing financial interests or personal relationships which have or could be perceived to have influenced the work reported in this article.
